# High uric acid exacerbates nonalcoholic steatohepatitis through NLRP3 inflammasome and gasdermin D-mediated pyroptosis

**DOI:** 10.1016/j.jbc.2025.110249

**Published:** 2025-05-19

**Authors:** Zixin Xu, Chenxi Tang, Xin Song, Zhening Liu, Jiaming Zhou, Qiaojuan Shi, Chaohui Yu, Chengfu Xu

**Affiliations:** 1 Department of Gastroenterology, The First Affiliated Hospital, Zhejiang University School of Medicine, Hangzhou, China; 2 Zhejiang Provincial Key Laboratory of Laboratory Animals and Safety Research, Hangzhou Medical College, Hangzhou, China

**Keywords:** uric acid, GLUT9, GSDMD, pyroptosis, NASH

## Abstract

Hyperuricemia is independently associated with an increased risk of nonalcoholic steatohepatitis (NASH), but the underlying mechanisms responsible for this association remain unclear. We first analyzed the association between intrahepatic UA levels and gasdermin D (GSDMD)-mediated pyroptosis *in vivo* and *in vitro*. We subsequently generated hepatic-specific glucose transporter 9 (GLUT9)-knockout mice and GSDMD knockout (GSDMD^−/−^) mice to explore the role of intrahepatic UA in GSDMD-induced pyroptosis in NASH. We found that high intrahepatic UA levels were positively related to GSDMD-mediated pyroptosis in NASH mice. The inhibition of hepatic UA production by allopurinol alleviated hepatic inflammation and GSDMD-mediated pyroptosis in NASH mice. Hepatic-specific knockout of Glut9 significantly decreased intrahepatic UA levels, attenuated NOD-like receptor family pyrin domain containing 3 (NLRP3)-Caspase-1-GSDMD-mediated pyroptosis in hepatocytes, and ameliorated hepatic inflammation and fibrosis in different mouse models of NASH. Further experiments revealed that inhibiting the NLRP3/Caspase-1/GSDMD pathway obviously blocked UA-induced pyroptosis and inflammation in hepatocytes. Additionally, GSDMD deficiency markedly reversed hepatic inflammation and fibrosis in NASH mice. In conclusion, our results showed that high UA could induce NLRP3-Caspase1-GSDMD-mediated pyroptosis, thereby aggravating NASH in mice.

Nonalcoholic steatohepatitis (NASH), an advanced stage of nonalcoholic fatty liver disease (NAFLD), is characterized by excessive fat accumulation, inflammation, and fibrosis (1). NASH is increasingly recognized as a leading etiology of cirrhosis, hepatocellular carcinoma, and liver-related mortality (2). However, the mechanisms involved in NASH pathogenesis remain unclear.

Pyroptosis is a specialized form of programmed cell death characterized by progressive cellular swelling, membrane rupture, and the release of cellular contents, which triggers a significant inflammatory response (3). In the canonical inflammatory caspase-mediated pyroptotic pathway, the NOD-like receptor family pyrin domain containing 3 (NLRP3) inflammasome acts as a sensor that activates downstream caspase-1. This activation leads to the cleavage of gasdermin D (GSDMD), producing the pore-forming N-terminus of GSDMD (GSDMD-N), which induces pyroptosis (4, 5). Pyroptosis predominantly occurs in macrophages, monocytes, and dendritic cells and has also been described in hepatocytes (6). GSDMD is widely considered an inflammatory caspase-mediated pore-forming effector protein during pyroptotic cell death, and increases in NLRP3-GSDMD levels have been observed in patients with NASH (7, 8, 9). However, the mechanism by which pyroptosis is involved in the development of NASH is still unknown.

A growing body of evidence indicates that uric acid (UA) plays an important role in the development of NAFLD. Elevated serum UA levels are related to an increased risk of both prevalent and incident NAFLD (10, 11, 12). Xanthine oxidase (XO) is a crucial enzyme for UA production, and allopurinol (an XO inhibitor) has been shown to affect the progression of NAFLD (13, 14, 15). UA has been reported to activate the NLRP3 inflammasome and regulate hepatic steatosis and insulin resistance in an NLRP3 inflammasome-dependent manner (16, 17). Glucose transporter 9 (GLUT9) acts as a key UA transporter and mediates the uptake of UA in hepatocytes (18). Hepatic-specific knockout of Glut9 significantly decreased the intrahepatic content and ameliorated diet-induced NAFLD in mice (19). However, the underlying mechanism by which GLUT9 regulates UA in NASH remains unclear.

In this study, we investigated the role of UA in hepatocyte pyroptosis and the pathogenesis of NASH. Our study revealed that intrahepatic UA is a novel driver of NLRP3-Caspase-1-GSDMD-mediated hepatocyte pyroptosis in NASH.

## Results

### Elevated intrahepatic UA contents are associated with NLRP3-GSDMD-mediated pyroptosis in NASH

**Figure 1:**
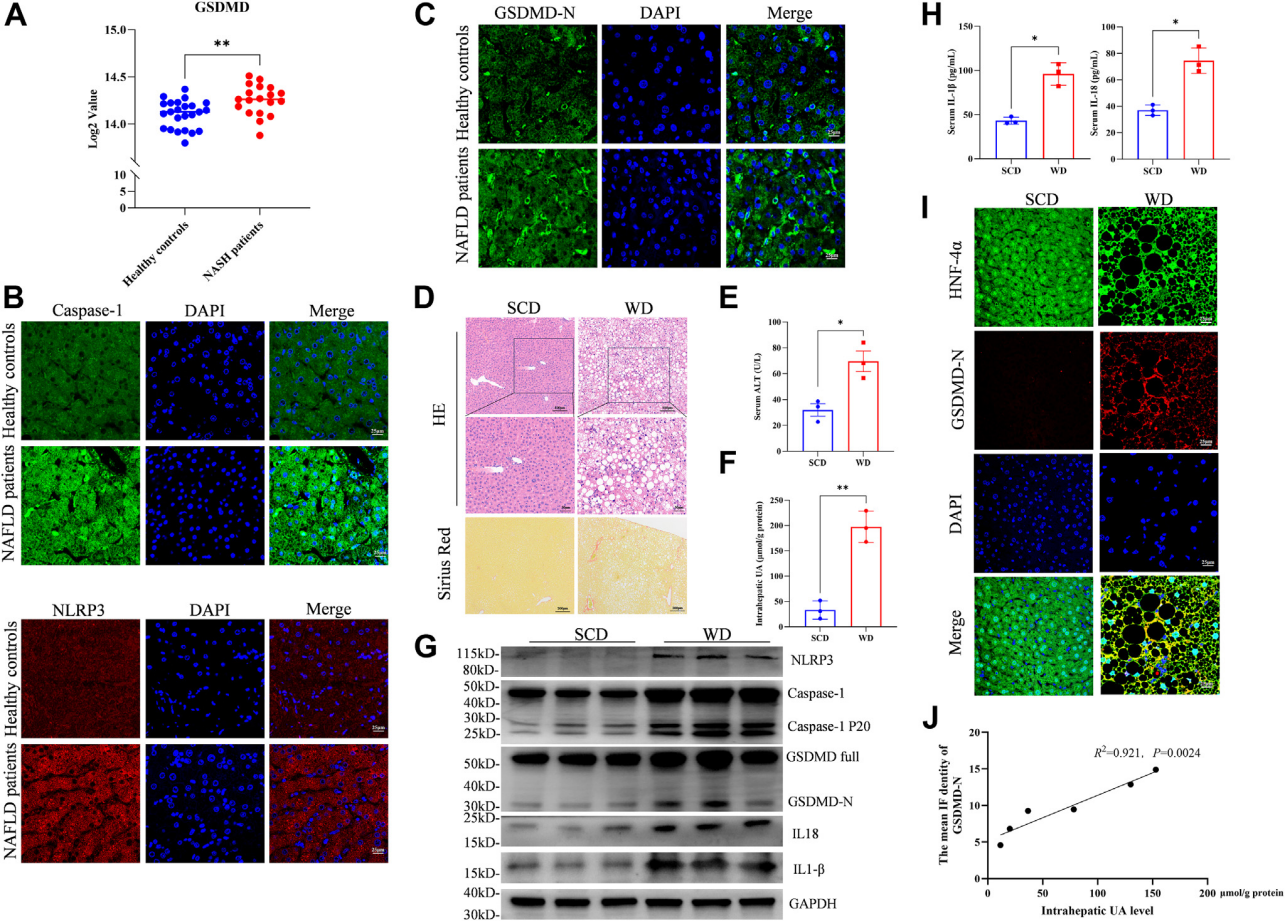
**Intrahepatic UA is associated with NLRP3-GSDMD-mediated pyroptosis in NASH.***A*, database analysis of *GSDMD* expression in livers from healthy controls (*n* = 24) and patients with NASH (*n* = 19). *B*, immunofluorescence staining showing the expression of NLRP3 and Caspase-1 in liver samples from healthy controls and NASH patients (*red*: NLRP3; *green*: Caspase-1; *blue*: DAPI). *C*, immunofluorescence staining was used to determine the expression of GSDMD in the liver tissues of healthy individuals (*n* = 3) and patients with NAFLD (*n* = 3). *D*, representative images of H&E and Sirius Red staining of liver tissues from SCD- and WD-fed mice (scan: 100×). *E*, serum ALT levels of SCD (*n* = 3) and WD (*n* = 3) mice. *F*, intrahepatic UA levels in SCD (*n* = 3) and WD (*n* = 3) mice. *G*, Western blot analyses of molecules related to NLRP3-GSDMD-induced pyroptosis. *H*, secreted forms of IL-1β and IL-18 in serum. *I*, expression of GSDMD-N in hepatocytes from SCD- and WD-fed mice (Scan: 630×). *J*, correlation analysis of the intrahepatic UA concentration and the immunofluorescence density of GSDMD-N (*n* = 6). The data are displayed as the means ± SDs. ∗*p* < 0.05, ∗∗*p* < 0.01 vs the SCD group.

Our previous study revealed that high UA levels regulate hepatic steatosis and insulin resistance through an NLRP3 inflammasome-dependent mechanism (16). Recently, accumulating evidence has shown that the activation of the NLRP3 inflammasome can induce pyroptosis (20). We analyzed the publicly available Gene Expression Omnibus database (GEO: GSE89632) and found that the expression of hepatic *GSDMD* was significantly increased in patients with NASH compared to healthy individuals (Fig. 1*A*). We found that the protein expression levels of NLRP3 and caspase-1 were significantly increased in the livers of patients with NAFLD (Fig. 1*B*). In addition, the GSDMD-N protein level was also elevated in liver samples from NAFLD patients compared with those from healthy controls (Fig. 1*C*).

To examine the potential relationship between UA and NASH in mice, we established a WD-fed mouse model of NASH. Histological analyses, including hematoxylin and eosin (H&E) and Sirius Red staining showed significant hepatic steatosis, necroinflammation, and fibrosis in the WD-fed mice, confirming the successful establishment of the NASH model (Fig. 1*D*). We found that WD-fed mice presented significantly higher serum ALT levels and intrahepatic UA levels than SCD-fed mice did (Fig. 1, *E* and *F*). Furthermore, the protein levels of key molecules associated with the classical pyroptosis pathway—such as NLRP3, full length and cleaved form of Caspase-1 and GSDMD, along with the cleaved forms of interleukin-1β (IL-1β) and interleukin-18 (IL-18)—were greatly increased in the livers of WD-fed mice (Fig. 1*G*). The serum levels of IL-1β and IL-18 were also significantly elevated in WD-fed mice (Fig. 1*H*). Notably, there were no significant differences in apoptosis or necroptosis between the SCD and the WD groups (Fig. S1*A*).

Hepatocytes are the main source of UA production in the liver. We found that GSDMD-N expression was markedly upregulated in the hepatocytes of WD-fed mice compared with those of SCD-fed mice (Fig. 1*I*). More importantly, we also identified a significant positive correlation between the increase in the intrahepatic UA level and the increase in the GSDMD-N protein level in hepatocytes (Fig. 1*J*). These results suggest a positive association between intrahepatic UA content and NLRP3-GSDMD-mediated pyroptosis in NASH patients.

### Inhibiting UA production significantly alleviated WD-induced hepatocyte pyroptosis and hepatic inflammation in mice

**Figure 2:**
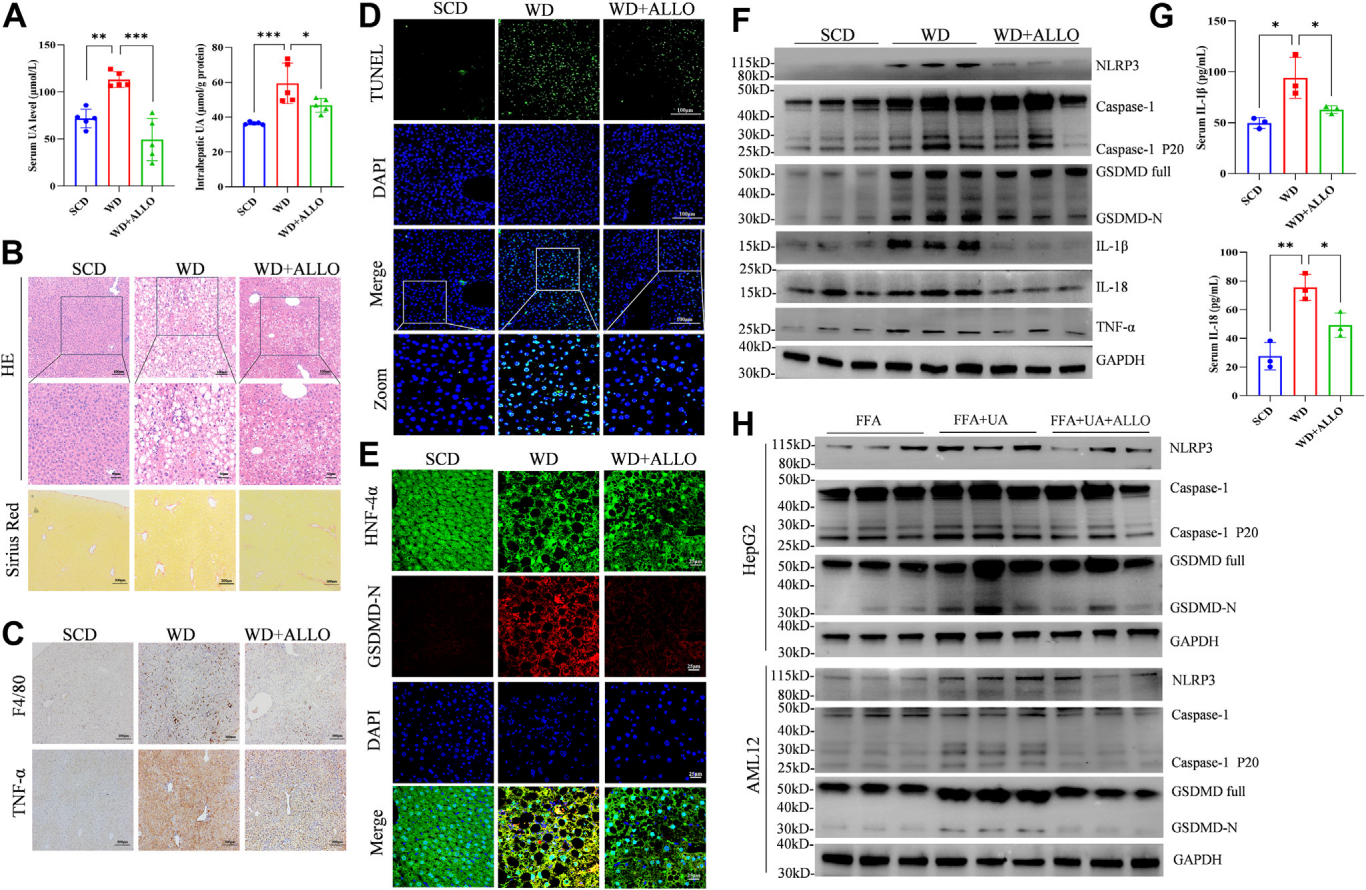
**Lowering UA reduced inflammation in NASH mice by increasing hepatocyte pyroptosis.***A*, intrahepatic UA levels in SCD, WD and WD + allopurinol (WD + ALLO) mice (*n* = 5 in each group). *B*, representative images of H&E and Sirius Red staining of liver tissues from SCD, WD and WD + allopurinol mice (*n* = 5). *C*, immunohistochemical staining of F4/80- and TNF-α-positive areas in the liver (scan: 100×). *D*, TUNEL staining of SCD, WD and WD + allopurinol mice (scan: 100×). *E*, double immunofluorescence staining was used to analyze the expression of GSDMD-N in hepatocytes in the liver tissues of mice (*red*: GSDMD-N; *green*: HNF-4α; *blue*: nucleus; *yellow*: overlay, scan: 630×). *F*–*H*, analyses of the expression of molecules related to NLRP3-GSDMD-induced pyroptosis *in vivo* and *in vitro* (*n* = 3). The data are displayed as the means ± SDs. ∗*p* < 0.05, ∗∗*p* < 0.01.

To determine whether UA can influence hepatocyte pyroptosis in NASH, we administered allopurinol in the drinking water to inhibit UA production in mice. Our results indicated that allopurinol treatment significantly decreased both the serum and intrahepatic UA levels in WD-fed mice (Fig. 2*A*). Additionally, allopurinol treatment led to a marked decrease in body weight, liver weight, and the serum levels of ALT, aspartate transaminase (AST) and triglyceride (TG) in WD-fed mice (Fig. S1, *B* and *C*). Moreover, inhibiting UA with allopurinol significantly ameliorated hepatic steatosis, reduced the number of activated hepatic stellate cells (Figs. 2*B* and S1*D*), and decreased the infiltration of F4/80, tumor necrosis factor-α (TNF-α), and terminal deoxynucleotidyl transferase-mediated dUTP nick-end labeling (TUNEL)-positive cells in the livers of WD-fed mice (Fig. 2, *C* and *D* and Fig. S1*E*). Allopurinol treatment also significantly reduced the levels of key proteins involved in the pyroptosis pathway, including NLRP3, full length of Caspase-1 and GSDMD, Cleaved Caspase-1, GSDMD-N, IL-1β, and IL-18 (Fig. 2, *E* and *F*) as well as the secreted forms of IL-1β and IL-18 (Fig. 2*G*). *In vitro*, UA significantly increased the protein levels of pyroptosis-related molecules in free fatty acid (FFA)-stimulated hepatocytes (Fig. 2*H*). However, allopurinol treatment significantly attenuated the UA-induced upregulation of pyroptosis-related molecules in AML12 and HepG2 cells (Fig. 2*H*). These findings showed that inhibiting UA production with allopurinol effectively alleviated WD-induced hepatocyte pyroptosis and hepatic inflammation in mice.

### Hepatic-specific knockout of Glut9 improves NASH-related inflammation

To further investigate the role of UA in NASH, we fed mice a WD and injected them with CCl_4_ to establish another NASH mouse model (Fig. S2*A*) according to previous work (21). GLUT9, which is expressed mainly in the liver, mediates the uptake of UA in hepatocytes (22, 23). We generated Glut9^HKO^ mice and Glut9^fl/fl^ mice as previously described (19).

**Figure 3:**
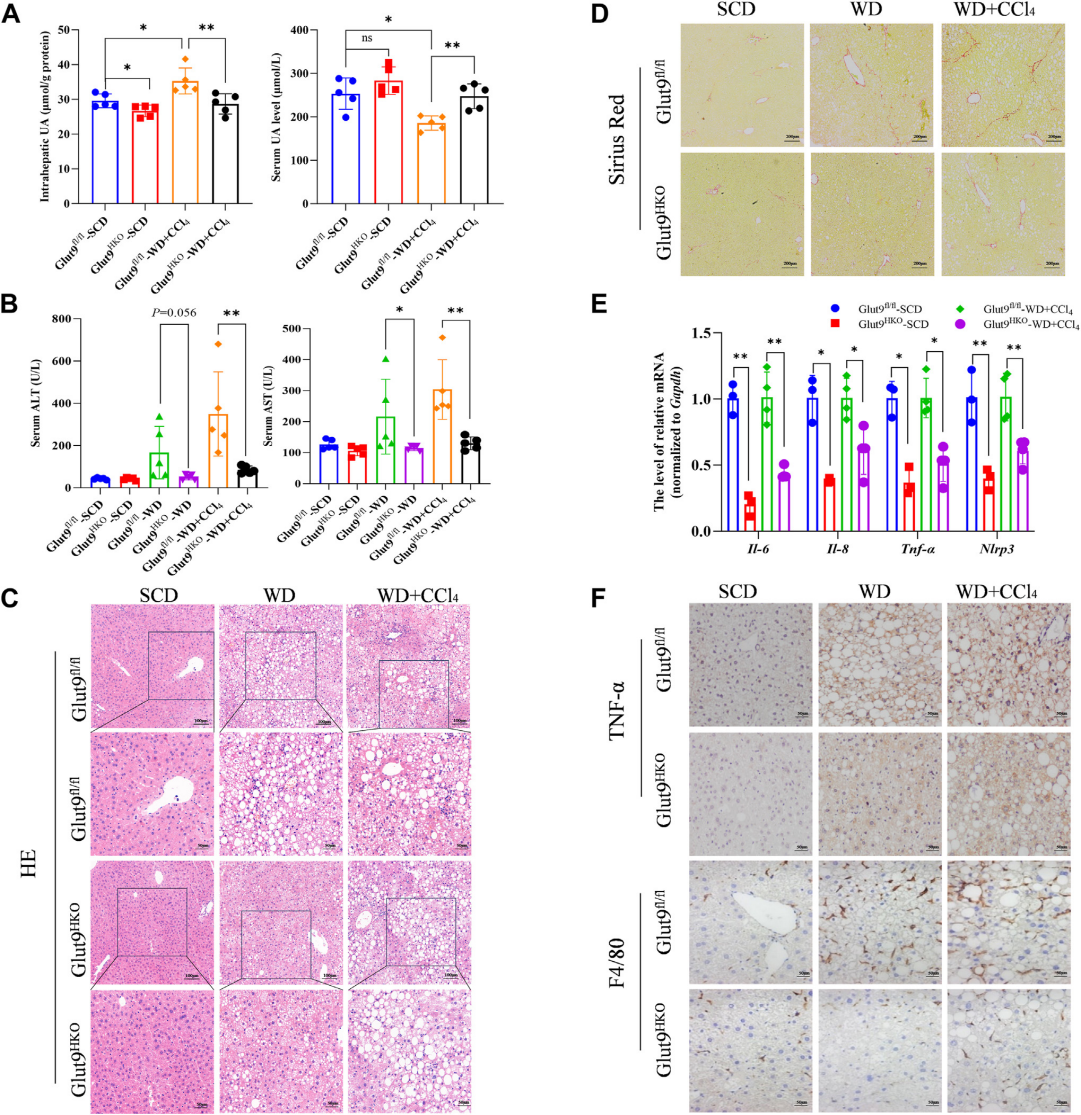
**Hepatic-specific knockout of Glut9 improves NASH-related inflammation.***A*, UA levels in the serum and liver of Glut9^HKO^ mice and Glut9^fl/fl^ mice (n = 5 in each group). *B*, serum ALT and AST levels of Glut9^HKO^ and Glut9^fl/fl^ mice in the SCD, WD and WD + CCl_4_ groups (*n* = 5 in each group). *C* and *D*, H&E and Sirius Red were used to visualize the pathology of Glut9^HKO^ and Glut9^fl/fl^ mice in the SCD group, WD group and WD + CCl_4_ group (*n* = 5 in each group). *E* and *F*, the expression of inflammation-related genes was tested by QPCR (*n* = 3) and IHC (*n* = 4). The data are displayed as the means ± SDs. ∗*p* < 0.05, ∗∗*p* < 0.01.

As expected, the UA level was lower in the liver but greater in the serum of Glut9^HKO^ mice than in that of Glut9^fl/fl^ control mice (Fig. 3*A*). Interestingly, there was a significant difference in the ratio of body weight to liver weight, as well as in the ITT and GTT results, between the Glut9^HKO^ and the Glut9^fl/fl^ mice (Fig. S2, *B*–*D*), although there was no difference in the expression of Glut9 in different mouse models (Fig. S2, *E* and *F*). Consistent with these findings, Glut9^HKO^ mice exhibited ameliorated WD + CCl_4_-induced hepatic steatosis and fibrosis (Figs. 3, *C* and *D*, S3, *A*–*C*), along with reduced serum ALT and AST levels (Fig. 3*B*). We also found that hepatic-specific knockout of Glut9 led to a decrease in the mRNA expression of inflammation-related genes, including *Il-6, Il-8, Tnf-α*, and *Nlrp3* (Fig. 3*E*). Furthermore, hepatic-specific Glut9 knockout inhibited the increase in F4/80 and TNF-α immunoreactivity induced by WD + CCl_4_ treatment in mice (Fig. 3*F*). These findings indicated that hepatic-specific knockout of Glut9 obviously decreased intrahepatic UA levels and attenuated WD + CCl_4_-induced liver inflammation and fibrosis in mice.

### Hepatic-specific knockout of Glut9 attenuates GSDMD-induced pyroptosis by decreasing the intrahepatic UA content in NASH

**Figure 4:**
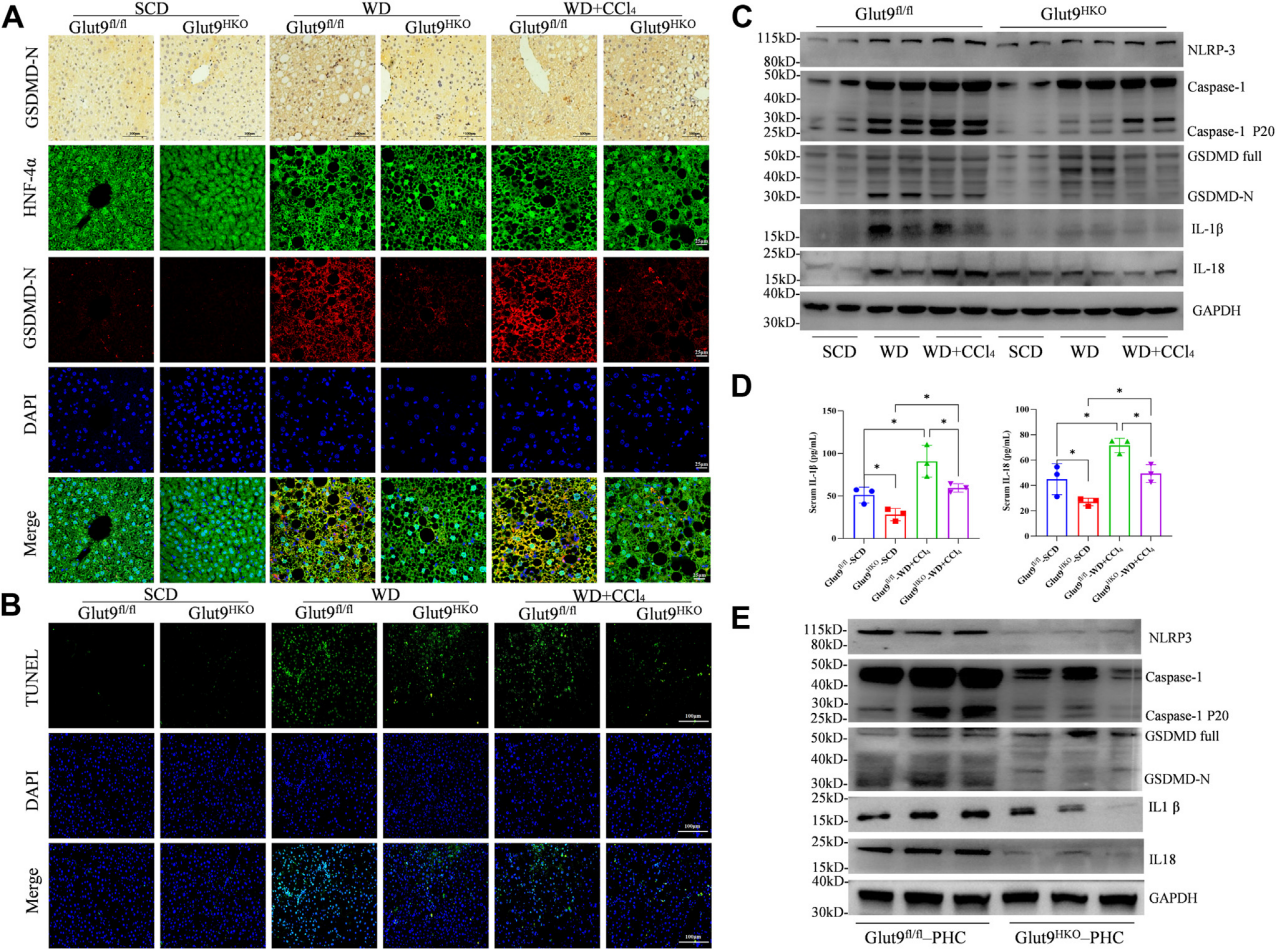
**Hepatic-specific knockout of Glut9 attenuates GSDMD-induced pyroptosis by decreasing intrahepatic UA elevation in NASH.***A*, IF and IHC analysis of GSDMD-N expression in the livers of Glut9^HKO^ and Glut9^fl/fl^ mice (*n* = 4). *B,* TUNEL staining was used to evaluate cell death in the livers of Glut9^HKO^ and Glut9^fl/fl^ mice*.C*, the expression of proteins involved in NLRP3-GSDMD-mediated pyroptosis was tested *via* western blotting. *D*, the serum levels of IL-1β and IL-18 were measured *via* ELISA. *E*, primary hepatocytes were isolated from Glut9^HKO^ and Glut9^fl/fl^ mice. Western blot analyses of NLRP3-GSDMD-mediated pyroptosis in primary hepatocytes from Glut9^HKO^ and Glut9^fl/fl^ mice (*n* = 3 in each group). The data are displayed as the means ± SDs. ∗*p* < 0.05.

Given that inhibiting UA production by allopurinol significantly alleviated WD-induced hepatocyte pyroptosis in mice, we investigated the impact of the hepatic-specific knockout of Glut9 on pyroptosis and other types of cell death. Our results indicated that the density of GSDMD-N protein in hepatocytes, which was increased by either WD or WD + CCl_4_ treatment, was notably decreased in Glut9^HKO^ mice (Fig. 4*A*). Moreover, we found that hepatic-specific knockout of Glut9 significantly decreased WD- or WD + CCl_4_-induced cell death, as reflected by fewer pyroptotic cells observed (Fig. 4*B*). In addition, the levels of GSDMD-mediated pyroptosis were markedly lower in the livers of Glut9^HKO^ mice subjected to either WD or WD + CCl_4_ treatment than in those of control mice (Fig. 4*C*) as were the levels of the secreted forms of IL-1β and IL-18 (Fig. 4*D*). Additionally, primary hepatocytes derived from Glut9^HKO^ mice presented lower expression of NLRP3, full-length and cleaved form of Caspase-1, GSDMD and GSDMD-N as well as IL-1β and IL-18 than those derived from Glut9^fl/fl^ mice following FFA and UA stimulation or UA treatment alone (Figs. 4*E* and S4*A*). Furthermore, these primary hepatocytes from Glut9^HKO^ mice presented reduced expression of inflammatory genes and decreased accumulation of lipid droplets in response to UA treatment (Fig. S4, *B*–*D*). There was also a notable decrease in the activation of hepatic stellate cells in these Glut9^HKO^ hepatocytes (Fig. S4*E*).

These findings imply that hepatic-specific knockout of Glut9 may reduce intrahepatic UA levels, which could subsequently alleviate liver GSDMD-induced hepatocyte pyroptosis and delay the progression of NASH both *in vivo* and *in vitro*.

### Hepatic-specific knockout of Glut9 attenuates MCD diet-induced NASH by mitigating hepatocyte pyroptosis

**Figure 5:**
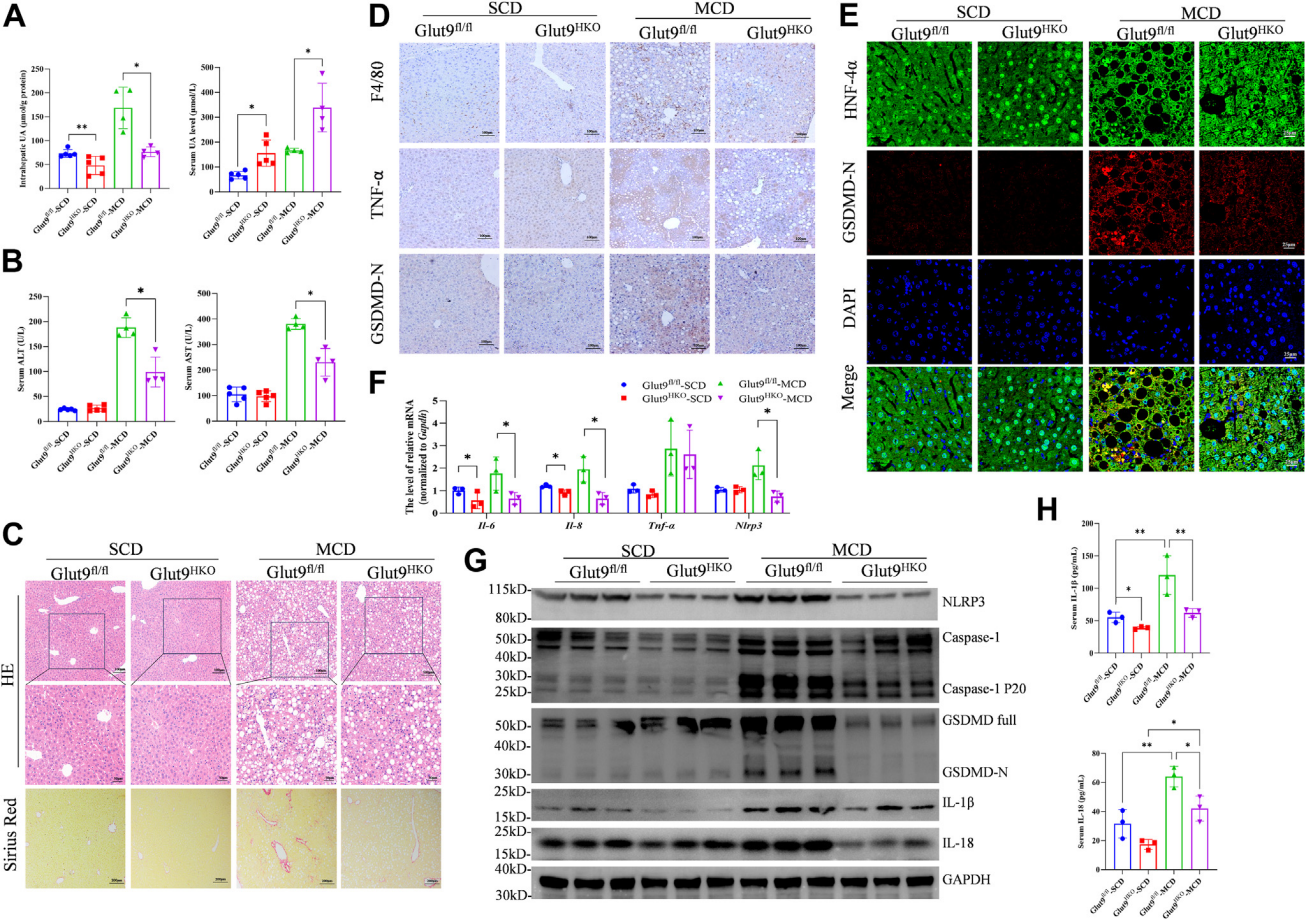
**Hepatic Glut9 deletion attenuates MCD-induced NASH by mitigating hepatocyte pyroptosis.***A*, serum and liver UA levels in Glut9^HKO^ and Glut9^fl/fl^ mice after SCD (*n* = 5) or MCD diet feeding (*n* = 4). One mouse died after MCD diet feeding. *B*, serum levels of ALT and AST in Glut9^HKO^ and Glut9^fl/fl^ mice fed an SCD (*n* = 5) or an MCD diet (*n* = 4). *C*, representative images of H&E and Sirius Red staining of liver tissues from Glut9^HKO^ and Glut9^fl/fl^ mice fed an SCD (*n* = *5*) or MCD diet (*n* = 4). *D*, IHC analysis of F4/80 and TNF-α as well as GSDMD-N expression in the livers of Glut9^HKO^ and Glut9^fl/fl^ mice fed an SCD or MCD diet. *E*, IF analysis of GSDMD-N in hepatocytes from Glut9^HKO^ and Glut9^fl/fl^ mice fed an SCD or MCD diet. *F*, the mRNA expression of inflammation-related genes, including *Nlrp3*, *Tnf-α*, *Il-6 and Il-8*. *G*, Western blot analyses of NLRP3-GSDMD-mediated pyroptosis in liver tissues from Glut9^HKO^ and Glut9^fl/fl^ mice. *H*, the serum levels of IL-1β and IL-18 were measured *via* ELISA. The data are displayed as the means ± SDs. ∗*p* < 0.05, ∗∗*p* < 0.01.

We next examined the role of hepatic-specific knockout of Glut9 in another mouse model of NASH induced by an MCD diet. Consistent with the above findings, intrahepatic UA levels were significantly lower, but serum UA levels were obviously greater in Glut9^HKO^ mice than in Glut9^fl/fl^ control mice following 6 weeks of MCD diet feeding (Fig. 5*A*). Moreover, hepatic-specific deletion of Glut9 resulted in decreased serum ALT and AST levels (Fig. 5*B*), decreased TG content in the serum and liver (Fig. S5*A*) and improved steatosis and fibrosis (Figs. 5*C* and S5, *B*–*D*). Similar to those in the WD or WD + CCl_4_ treatment groups, fewer F4/80- or TNF-α-positive cells were observed in the livers of Glut9^HKO^ mice fed the MCD diet (Fig. 5*D*). Notably, immunobiological staining revealed fewer GSDMD-positive hepatocytes in Glut9^HKO^ mice than in Glut9^fl/fl^ control mice following the MCD diet (Fig. 5, *D* and *E*). Compared with the Glut9^fl/fl^ control mice, the Glut9^HKO^ mice presented a reduced mRNA expression of inflammation-related factors (Fig. 5*F*) and decreased activation of NLRP3 and Caspase-1, GSDMD-N, IL-1β and IL-18, as reflected by the lower expression levels of these molecules in these mice (Fig. 5, *G* and *H*). These findings indicated that hepatic-specific deletion of Glut9 could indeed relieve GSDMD-mediated pyroptosis and NASH by reducing the intrahepatic UA content.

### Inhibition of pyroptosis alleviates UA-induced hepatocyte injury *in vitro*

**Figure 6:**
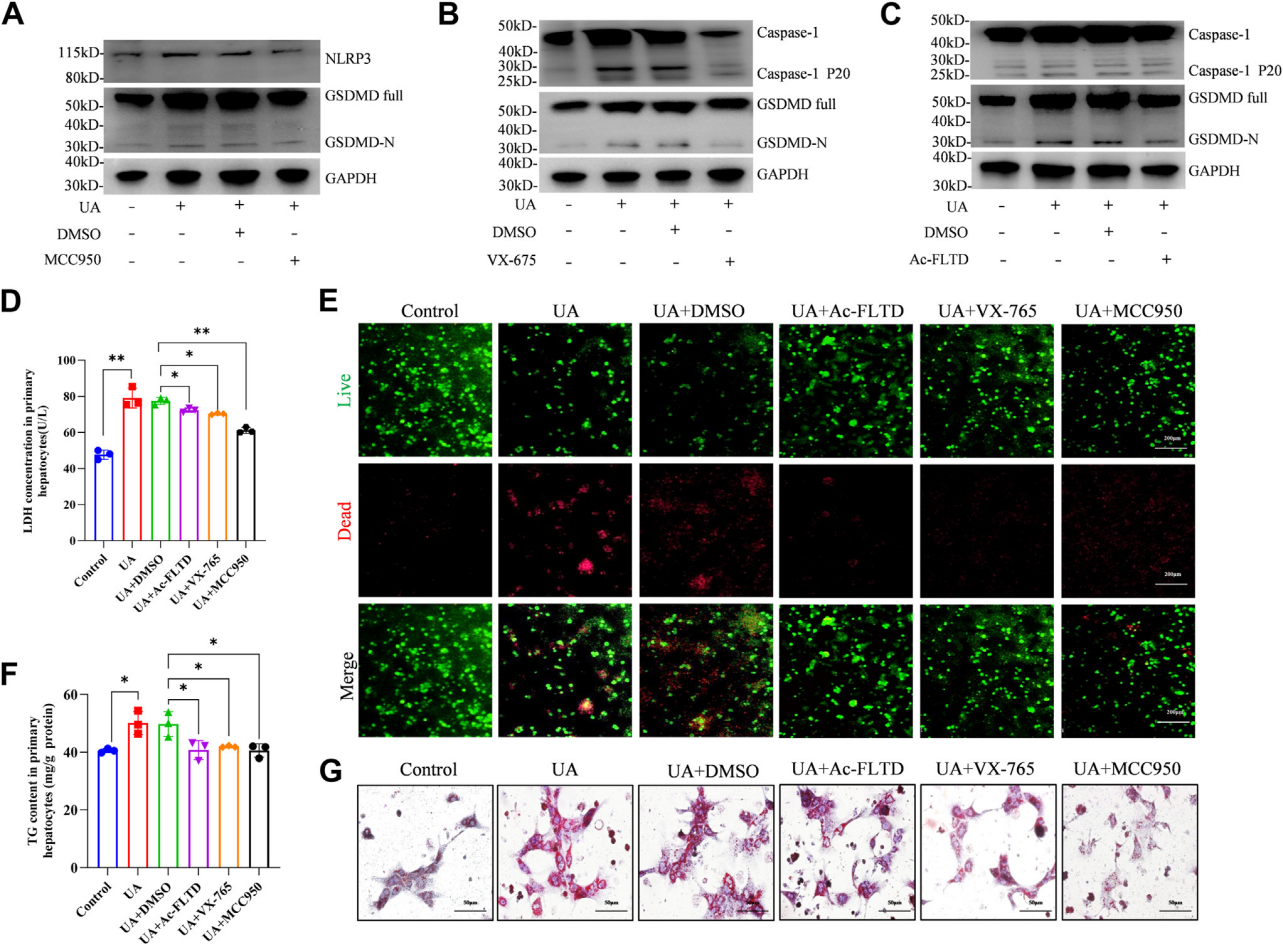
**Inhibition of pyroptosis alleviates UA-induced hepatocyte injury *in vitro***. *A*–*C*, different inhibitors targeting NLRP3, Caspase-1 and GSDMD were used to treat primary hepatocytes. Western blotting was also conducted to test the expression of NLRP3, Caspase-1 and GSDMD. *D* and *E*, cell death was evaluated by LDH concentration and the number of live or dead hepatocytes after different treatments. *F*, TG contents of primary hepatocytes after different treatments. *G*, ORO analysis of the effects of various inhibitors on UA-induced lipid accumulation. The data are displayed as the means ± SDs. ∗*p* < 0.05, ∗∗*p* < 0.01.

Considering that UA enhances GSDMD-mediated pyroptosis and exacerbates NASH in mice, we explored whether inhibiting GSDMD-induced pyroptosis could reverse these effects. First, we treated primary hepatocytes from wild-type mice with an NLRP3 inhibitor (MCC950), a Caspase-1 inhibitor (VX-765), or a gasdermin D-derived inhibitor (Ac-FLTD-CMK). Even after exposure to UA, these inhibitors not only significantly reduced the expression of NLRP3 and Caspase-1 but also significantly inhibited the activation of intracellular GSDMD induced by high levels of UA (Fig. 6, *A*–*C*). Moreover, the inhibitors significantly inhibited UA-induced cell death, which was reflected by the reduced release of LDH in the cell supernatant and a lower proportion of dead and viable primary hepatocytes and AML12 cells (Fig. 6, *D* and *E* and Fig. S6, *A*). The application of these inhibitors also significantly alleviated the UA-induced increase in the number of intracellular lipid droplets and TG content in hepatocytes (Fig. 6, *F* and *G* and Fig. S6*, B*). These findings indicate that inhibiting pyroptosis could be an effective therapy for alleviating UA-induced hepatocyte injury in NASH patients.

### Deletion of GSDMD alleviates liver inflammation and fibrosis in NASH mice

**Figure 7:**
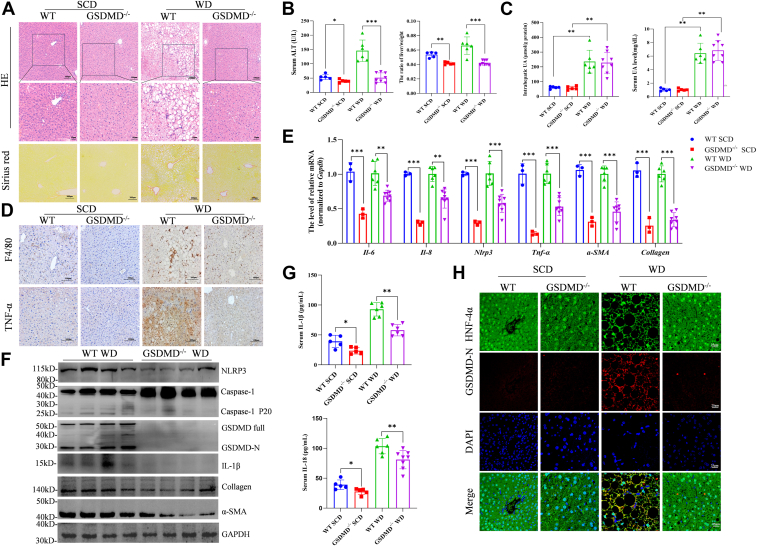
**GSDMD deficiency alleviates liver inflammation and fibrosis caused by high UA in NASH.***A*, representative images of H&E and Sirius Red staining in GSDMD^−/−^ (SCD, *n* = 5; WD, *n* = 8) and WT (SCD, *n* = 5; WD, *n* = 6) mice. *B*, ratios of liver weight to weight and the serum level of ALT in the above-described mice. *C*, the levels of UA in the serum and liver of GSDMD^−/−^ mice and WT mice fed a WD or SCD. *D*, IHC analysis of F4/80 and TNF-α in the livers of GSDMD^−/−^ mice and WT mice fed a SCD or WD. *E*, the mRNA expression of inflammation-related genes, including *Nlrp3, Tnf-α, Il-6* and *Il-8*, as well as fibrosis-related genes. *F*, Western blot analysis of NLRP3-GSDMD-mediated pyroptosis and fibrosis in liver tissues from GSDMD^−/−^ mice (*n* = 4) and WT mice (*n* = 4) fed a WD. *G*, the serum levels of IL-1β and IL-18 were tested *via* ELISA. *H*, IF analysis of GSDMD-N in hepatocytes from GSDMD^−/−^ mice and WT mice fed a SCD or WD. The data are displayed as the means ± SDs. ∗*p* < 0.05, ∗∗*p* < 0.01, ∗∗∗*p* < 0.0001.

These findings indicate that UA can induce GSDMD-dependent pyroptosis, which contributes to NASH progression. The exploration of GSDMD deletion as a therapeutic strategy is promising. After the mice were fed a WD, we observed that the GSDMD^−/−^ mice exhibited a reduced severity of hepatic steatosis and fibrosis (Fig. 7*A*). Furthermore, the absence of GSDMD significantly attenuated WD-induced liver damage, as evidenced by decreased ALT levels and liver/body ratios (Figs. 7*B* and S7*A*). Our results also revealed that, compared with WT mice, GSDMD^−/−^ mice presented no significant difference in hepatic or serum UA levels following SCD or WD feeding (Fig. 7*C*). Moreover, hepatic or serum UA levels were significantly increased in WD-fed WT and GSDMD^−/−^ mice (Fig. 7*C*). Additionally, the expression levels of F4/80 and TNF-α in the livers of GSDMD^−/−^ mice were markedly lower than those in the livers of control mice, accompanied by similar changes in inflammation-related genes (Fig. 7, *D* and *E*). Furthermore, the protein expression of NLRP3, Caspase-1, GSDMD-N, IL-1β, and IL-18 was measured by Western blotting (Figs. 7, *F*–*H* and S7*B*). Notably, the number of activated hepatic stellate cells and the expression of collagen and α-SMA in the livers of the GSDMD^−/−^ mice were also lower than those in the livers of the WT mice (Figs. 7*E* and S7*C*).

Taken together, our findings suggest that genetic ablation of GSDMD, an executive molecule involved in pyroptosis, effectively ameliorates the progression of NASH with high UA levels.

## Discussion

In this study, we present preliminary evidence establishing a connection between UA and GSDMD-mediated pyroptosis in NASH. The induction of GSDMD-mediated pyroptosis was found to coincide with elevated intrahepatic UA levels in NASH mice. Moreover, high intrahepatic UA concentrations triggered NLRP3-induced activation of Caspase-1, leading to GSDMD cleavage and subsequent hepatocyte pyroptosis both *in vivo* and *in vitro*.

NASH represents an advanced stage of NAFLD characterized by steatosis, hepatocyte injury (ballooning), and inflammation, with or without fibrosis (24, 25, 26). The pathophysiology of NASH is complex and still largely elusive. A critical challenge lies in pinpointing the factors driving the shift from simple fatty liver disease to NASH. Therefore, we investigated the potential role of UA in NASH. Intriguingly, our findings revealed a significant elevation not only in serum UA but also in intrahepatic UA levels in NASH mice. These findings suggest a potential contributory role of UA in the progression of NASH.

Hepatocellular programmed cell death, which encompasses apoptosis, necroptosis, and the recently identified pyroptosis, is recognized as a critical initiator of NASH (27, 28). Pyroptosis, the most recently known mode of programmed cell death, occurs more rapidly than does apoptosis and coincides with the release of a greater quantity of proinflammatory factors (29, 30, 31). GSDMD-induced pyroptosis has been identified as a contributing factor (32) in the initiation and progression of various liver disorders, such as alcoholic hepatitis (33) and NAFLD/NASH(8). Our results revealed that NLRP3-induced GSDMD-mediated pyroptosis, but not apoptosis or necroptosis, was significantly increased in the livers of NASH model mice. In the canonical pyroptosis pathway, pathogen-associated molecular patterns (PAMPs) and damage-associated molecular patterns (DAMPs) typically stimulate the NLRP3 inflammasome, leading to Caspase-1 activation and subsequent GSDMD cleavage to trigger pyroptosis (34, 35). Our previous research revealed that UA acts as a DAMP and triggers the production of the NLRP3 inflammasome, thereby aggravating NAFLD (16). Therefore, we hypothesized that UA affects GSDMD-mediated pyroptosis in NASH. In support of this hypothesis, we detected a significant increase in intrahepatic UA in NASH model mice. Moreover, correlation analysis revealed a positive correlation between UA levels and GSDMD expression in hepatocytes from NASH mice. These results suggest that UA may indeed affect GSDMD-mediated pyroptosis through NLRP3.

Hyperuricemia is a metabolic disorder characterized by excessive UA production in the liver or impaired elimination of UA in the kidneys (36). UA is synthesized mainly in hepatocytes from purine nucleotides *via* different enzymatic reactions and is subsequently released into the bloodstream (37). Allopurinol is a medication that regulates UA production by inhibiting xanthine oxidase. This inhibition reduces the conversion of hypoxanthine and xanthine to UA, consequently lowering UA levels in the body. We used allopurinol treatment to reduce UA production and found that it significantly lowered UA levels in the serum and liver of NASH model mice. Additionally, allopurinol treatment alleviated hepatic inflammation and fibrosis. Notably, it markedly reduced the protein expression of proteins involved in NLRP3-Caspase-1-GSDMD-related pyroptosis as well as that of IL-1β and IL-18 both *in vivo* and *in vitro*. These results further revealed that UA influences NLRP3-GSDMD-induced pyroptosis in NASH.

Additionally, hepatocytes can take up UA from the bloodstream *via* various transporters, such as GLUT9 (23). As shown in a previous report, hepatic knockout of Glut9 inhibited diet-induced intrahepatic UA elevation and alleviated UA-induced steatosis in mice (19). According to our results, liver-specific knockout of Glut9 significantly alleviates hepatic inflammation, steatosis and fibrosis in NASH mice. Moreover, liver-specific knockout of Glut9 significantly alleviated the increase in hepatic UA levels and alleviated the increase in UA-induced NLRP3/Caspase-1/GSDMD-mediated hepatocyte pyroptosis in primary hepatocytes and NASH mice, ultimately leading to a reduction in pyroptosis in the NASH model.

In mouse models, hepatic GLUT9 knockout affects the GTT or ITT in MASH diet-fed mice, indicating the possibility that UA regulates glucose. Elevated systemic UA levels are associated with impaired insulin signaling and exacerbated hepatic steatosis (38, 39). In the liver, UA may interfere with gluconeogenesis and glycogen storage, processes crucial for maintaining glucose homeostasis (40). Moreover, UA has been reported to inhibit IRS1-AKT signaling and insulin-stimulated glucose uptake to induce insulin signaling impairment (41, 42). RBP4 has been reported to be involved in HUA-induced IR by inhibiting IRS/PI3K/AKT phosphorylation (43). We previously reported that UA regulates insulin resistance through an NLRP3 inflammasome-dependent mechanism and promotes the progression of NAFLD (16). However, further experiments are needed to explore the potential mechanism of glucose regulation through UA in the progression of MASH.

Inhibition of pyroptosis has shown promising applications in various diseases (44, 45, 46). Specifically, blocking key molecules such as NLRP3 and Caspase-1 could help attenuate the pathological processes associated with conditions such as NASH and other liver disorders (8, 47, 48, 49). In our study, inhibitors directed against NLRP3, caspase-1, and GSDMD effectively blocked hepatocyte pyroptosis induced by high UA concentrations. Furthermore, GSDMD knockout improved hepatic steatosis, the inflammatory response and fibrosis in NASH model mice. Consistent with these findings, the expression levels of NLRP3, the cleaved form of Caspase-1, IL-18, and IL-1β were markedly reduced in the GSDMD^−/−^ mice with NASH. Interestingly, UA levels did not differ between the WT and GSDMD knockout groups, regardless of whether they were fed a control diet or a WD. The level of intrahepatic UA was greater in the WD group than in the control group. These findings indicate that UA production was not affected by GSDMD knockout. These results indicate that high intrahepatic uric acid levels promote NASH through GSDMD-mediated pyroptosis.

There are limitations in this study. We detected increased expression of NLRP3 and Caspase-1 in patients with NASH *via* immunofluorescence, suggesting a potential role in NASH progression. A more reliable way to measure protein expression *via* Western blotting would be to confirm previous findings reported in the literature that the expression of NLRP3 and Caspase-1 was increased in patients with NAFLD (50, 51, 52). The level of serum UA in WD group mice was increased but not in WD + CCl4 group mice compared with that in SCD group mice. A possible explanation for the unexpected UA levels in WD + CCl4-treated mice is that UA may play different roles in different stages of NAFLD. Previous clinical studies reported that serum UA levels were significantly associated with the severity of hepatic steatosis and inflammation but not with advanced fibrosis in patients with NAFLD (53, 54, 55), indicating that UA may exert different effects on the progression of NAFLD. The potential mechanism needs further exploration. As one of the transporters for glucose and fructose, GLUT9 has relatively minimal activity in this regard (56, 57). Whether the functions of glucose and fructose transport are involved in the process of NAFLD remains a question worth exploring.

In conclusion, our study is the first to show that elevated UA can induce pyroptosis *via* the NLRP3-Caspase1-GSDMD pathway, potentially exacerbating NASH in mice. Consequently, targeting the NLRP3-GSDMD pathway may represent a potential therapeutic strategy for addressing high UA-induced NASH.

## Experimental procedures

### Human study

Liver biopsies from liver transplant donors who were healthy adults (*n* = 3) and from patients with NAFLD (*n* = 3) who underwent liver biopsies for suspected NASH were randomly selected from liver biopsy samples collected at the First Affiliated Hospital, Zhejiang University School of Medicine. All liver tissue samples from patients and healthy controls included in this study were approved by the Ethics Committee of the First Affiliated Hospital, Zhejiang University School of Medicine (no. 2023--0422), and all participants provided written informed consent. The human research conducted in the study strictly complies with the Declaration of Helsinki principles.

### Animal experiments

Male C57BL/6 mice (6–8 weeks old), hepatic-specific knockout Glut9 mice (Glut9^HKO^) and Glut9 flox mice (Glut9^fl/fl^) were used. Glut9^HKO^ mice were generated by crossing Glut9-floxed mice with albumin-cre mice as previously reported (19). GSDMD^−/−^ mice were a gift from Professor Ying Zhang’s laboratory (the First Affiliated Hospital, Zhejiang University School of Medicine).

To establish a NASH mouse model, we designed an experimental group according to a protocol described previously (21). The Glut9^fl/fl^ and Glut9^HKO^ mice were randomly assigned to three groups: the standard chow diet (SCD) group (*n* = 5), the WD group (*n* = 5) and the WD + CCl_4_ group (*n* = 5). The SCD group received an SCD and ordinary water. The WD group was fed a WD (Teklad, TD 120528) comprising 21.1% fat, 41% sucrose, and 1.25% cholesterol and high sugar solutions containing 23.1 g/L d-fructose (Sigma‒Aldrich, G8270) and 18.9 g/L d-glucose (Sigma‒Aldrich, F0127). The WD + CCl_4_ group received the same diet and water as the WD group. CCl_4_ (Sigma‒Aldrich, 28916--100 ml) diluted in corn oil was administered intraperitoneally at a dosage of 0.2 μl (0.32 μg)/g body weight weekly, commencing concurrently with the dietary regimen.

To evaluate the effect of allopurinol-mediated inhibition of UA production on NASH, wild-type (WT) mice were randomly divided into three groups and were fed an SCD, a WD, or a WD (*n* = 5) combined with allopurinol (120 mg/L) in their drinking water.

For the methionine and choline-deficient (MCD) diet model, Glut9^fl/fl^ and Glut9^HKO^ mice were randomly divided into two groups that were fed an SCD or MCD diet (A02082002BR, Research Diets, New Brunswick, NJ) (*n* = 5).

To explore the relationship between UA and GSDMD in NASH, WT mice (*n* = 11) and GSDMD^−/−^ mice (*n* = 13) were fed a SCD or WD for 12 weeks.

After 14 weeks of WD/WD + CCl_4_ or 6 weeks of MCD diet feeding, the mice were sacrificed, and blood and liver samples were collected at the indicated time points for further analysis. All animal experiments were performed according to guidelines approved by the Animal Care and Use Committee of Zhejiang Provincial Key Laboratory of Laboratory Animals and Safety Research, Hangzhou Medical College (approval no. ZJCLA-IACUC-20010182).

### Biochemical measurement

The concentrations of alanine aminotransferase (ALT), aspartate transaminase (AST), triglyceride (TG), and UA in the serum and lactate dehydrogenase in the supernatant, as well as the intrahepatic TG content, were measured with commercial kits (Nanjing Jiancheng Bioengineering Institute) according to the manufacturer’s instructions. The levels of UA in the cells and liver were measured with a UA assay kit (Bioassay Systems) and normalized to the total protein content. In accordance with our previous work (58), liver samples were weighed, homogenized in saline and then centrifuged to obtain the supernatant. The supernatant was subsequently used to measure the level of UA. The UA levels of each sample were normalized to the protein concentration and expressed as μmol/g protein.

### Immunofluorescence and immunohistochemistry analyses

Liver sections were fixed in a 4% paraformaldehyde solution and then embedded in paraffin for hematoxylin and eosin staining, Sirius red staining, immunohistochemistry analysis, immunofluorescence staining, and TUNEL staining. For immunohistochemistry and immunofluorescence analysis, the sections were generated as previously described (59). Representative images were captured *via* a Leica STELLARIS 5 confocal fluorescence microscope (Leica Microsystems). The relative antibodies and isotype controls used are listed in Table S1.

### Insulin tolerance tests (ITTs) and glucose tolerance tests (GTTs)

ITTs and GTTs were performed for mice fed an SCD or WD as previously described (16, 19). For ITTs, the mice were injected intraperitoneally with an insulin solution (1 mU/g body weight) following a 6-h fasting period. For the GTTs, the mice were injected intraperitoneally with glucose solution (1 mg/g body weight) after a 16-h fasting period. Blood glucose levels were measured *via* a Freestyle brand glucometer (LifeScan) from the tail tip.

### Enzyme-linked immunosorbent assay (ELISA)

The levels of IL-1β and IL-18 in the serum were measured *via* ELISA kits (ABclonal). The detection methods were performed according to the instructions of the manufacturers.

### Quantitative real-time PCR (qPCR)

Total RNA was extracted and subsequently reverse-transcribed into cDNA. qPCR was performed *via* a SYBR Green PCR Kit (Accurate Biotechnology Co, Ltd). The primers used in this research are listed in Table S2. All qPCR was repeated three times.

### Western blot

The experiments were conducted according to previous work (19)^.^ All the primary antibodies used in the study are listed in Table S1. The secondary antibody used was HRP-IgG (1:5000; Dakewe Biotech Co, Ltd).

### Isolation and culture of primary hepatocytes

Mouse primary hepatocytes (PHCs) were isolated from male C57BL/6 mice as previously described (58, 60). For PHC culture experiments, 500,000 cells per well were plated in collagen-coated 6-well or 12-well plates supplemented with 100 mg/ml streptomycin, 100 IU/ml penicillin, and 10% fetal bovine serum (FBS; Gibco).

### Live and dead cell stain

Using commercial kits (Invitrogen, R37601), we first prepared the medium with 1x stain buffer and transferred this medium into the cells for incubation for another 15 to 20 min at 20 to 25 °C. Finally, the primary hepatocytes were fixed and observed *via* confocal microscopy.

### Cell line culture and treatment

AML12 cells and HepG2 cells were purchased from ATCC (American Type Culture Collection) and used in this study. The cells were cultured in DMEM or RPMI-1640 medium supplemented with 10% FBS, 100 mg/ml streptomycin and 100 IU/ml penicillin. To establish a cellular model of NAFLD, the cells were treated with free fatty acids (FFAs) at a final concentration of 1 mM for 24 h and a 2:1 mixture of oleate and palmitate as we previously described. To further evaluate the effects of UA on the development of NAFLD, the cells were exposed to high concentrations (750 μmol/L) of UA for 24 h. Unless otherwise specified, the following concentrations of reagents were applied *in vitro*: MCC950 (50 μM), VX-765 (20 μM), and Ac-FLTD (10 μM).

### Oil red O (ORO) staining

The cells were subjected to ORO in accordance with the manufacturer’s protocols. The cell samples were imaged and recorded under a Leica microscope (Leica) following incubation with 200 μl of ORO working solution and washing three times with PBS.

### Statistical analysis

The data are presented as the mean ± standard deviation (SD). Comparisons between two groups were analyzed with an unpaired Student’s *t* test, and differences among three groups were analyzed by one-way ANOVA. Pearson’s correlation was used to evaluate the relationship between the mean fluorescence intensity of GSDMD-N and the level of intrahepatic UA in the mice. All the statistical analyses were performed *via* GraphPad Prism software (Inc.). A *p* value < 0.05 was considered to indicate statistical significance.

## Data availability

The data analyzed during this study are included in this published article. Additional supporting data are available from the corresponding authors upon reasonable request.

## Supporting information

This article contains supporting information.

## Conflict of interest

The authors declare that they have no conflicts of interest with the contents of this article.
