# Supplementary material for: High uric acid exacerbates nonalcoholic steatohepatitis through NLRP3 inflammasome and gasdermin D-mediated pyroptosis
Source: J Biol Chem. 2025 May 19;301(6):110249. doi: 10.1016/j.jbc.2025.110249 (PMC12205687; doi:10.1016/j.jbc.2025.110249)
Supplement: Supporting information figures [file mmc1.docx]

**Supplementary materials**

**High uric acid exacerbates nonalcoholic steatohepatitis through NLRP3 inflammasome and Gasdermin D-mediated pyroptosis**

Zixin Xu^1^, Chengxi Tang^1^, Xin Sun^1^, Zhening Liu^1^, Jiaming Zhou^1^, Qiaojuan Shi^2^,*, Chaohui Yu^1^,*, Chengfu Xu^1^,*

1 Department of Gastroenterology, the First Affiliated Hospital, Zhejiang University School of Medicine, Hangzhou 310003, China

2 Zhejiang Provincial Key Laboratory of Laboratory Animals and Safety Research, Hangzhou Medical College, Hangzhou 310063, China

**Supplementary Figures**

**Supplementary Figure 1**


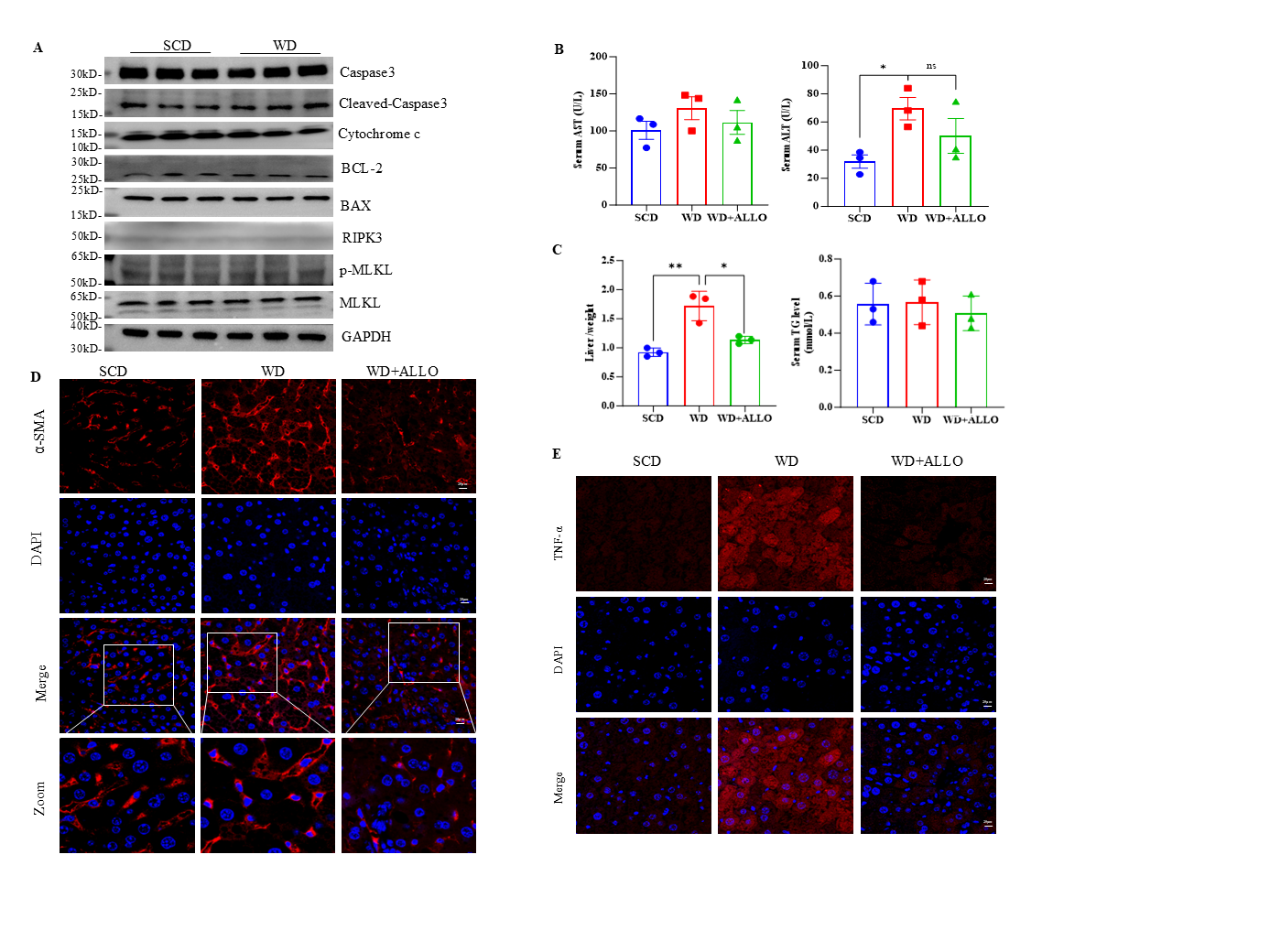


**Supplementary Figure 1 Inhibiting UA production significantly alleviated WD-induced hepatocyte injury and fibrosis**

A. The expression of necrosis and apoptosis related protein in SCD and WD group.

B. The level of ALT and AST in serum was decreased after allopurinol treatment.

C. The liver/weight ratio and TG in serum were tested in different group.

D. The activation of HSCs was identified by the area of α-smooth muscle actin(α-SMA) positive.

E. Immunofluorescence staining of TNF-α-positive areas in the liver (scan: 630×).

Data are representative of three independent experiments with similar results. **P*<0.05, ***P*< 0.01.

**Supplementary Figure 2**

**
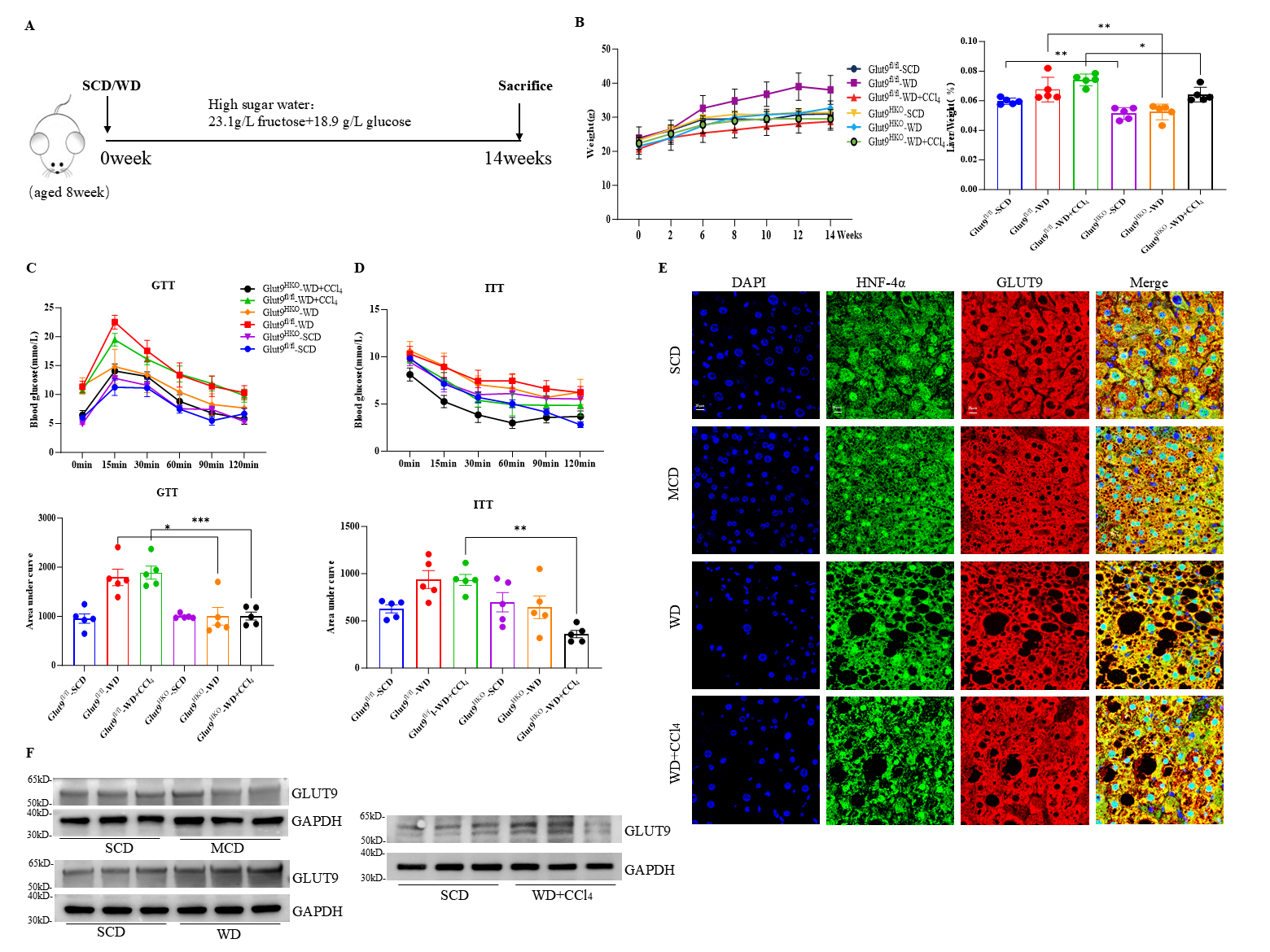
**

**Supplementary Figure 2 An establishment of NASH mouse model in Glut9^HKO^ and Glut9^fl/fl^ mice**

A. The establishment of a NASH mouse model

B. The curve of weight and liver/weight ratio in different group.

C-D. The GTT and ITT test were conducted in different group.

E-F. The expression of GLUT9 in NASH mice.

Data are representative of three independent experiments with similar results. **P*<0.05, ***P*< 0.01.

**Supplementary Figure 3**

**
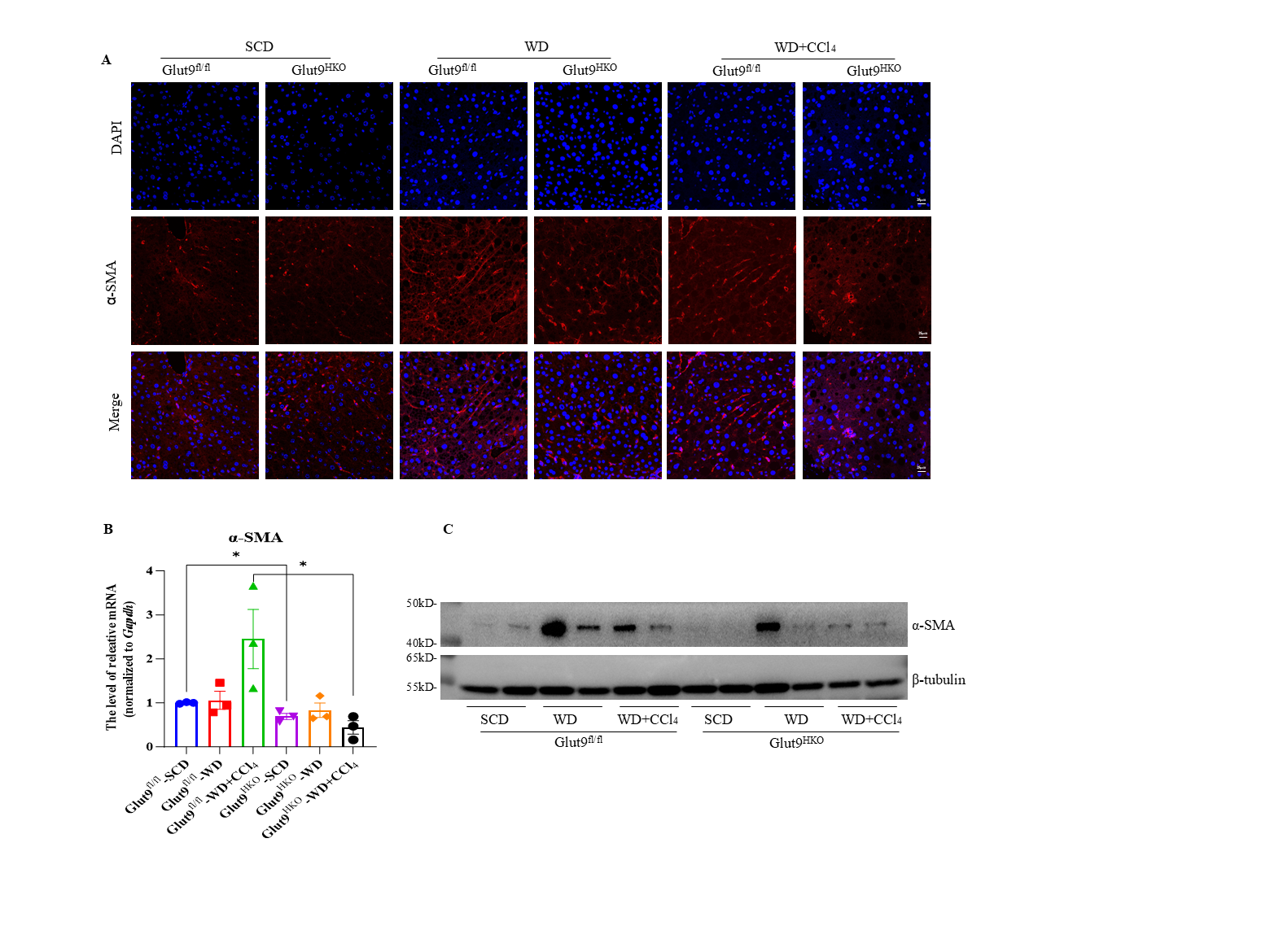
**

**Supplementary Figure 3 Glut9 liver specific knockout significantly alleviated the activation of HSCs in NASH mouse model**

A. The activation of HSCs was decreased in Glut9^HKO^ mice group than that of Glut9^fl/fl^ group mice whether SCD or WD/WD+CCl4 treatment.

B-C. The QPCR and Western blot showed a decreased expression of α-SMA in Glut9^HKO^ mice group.

Data are representative of three independent experiments with similar results. **P*<0.05.

**Supplementary Figure 4**

**
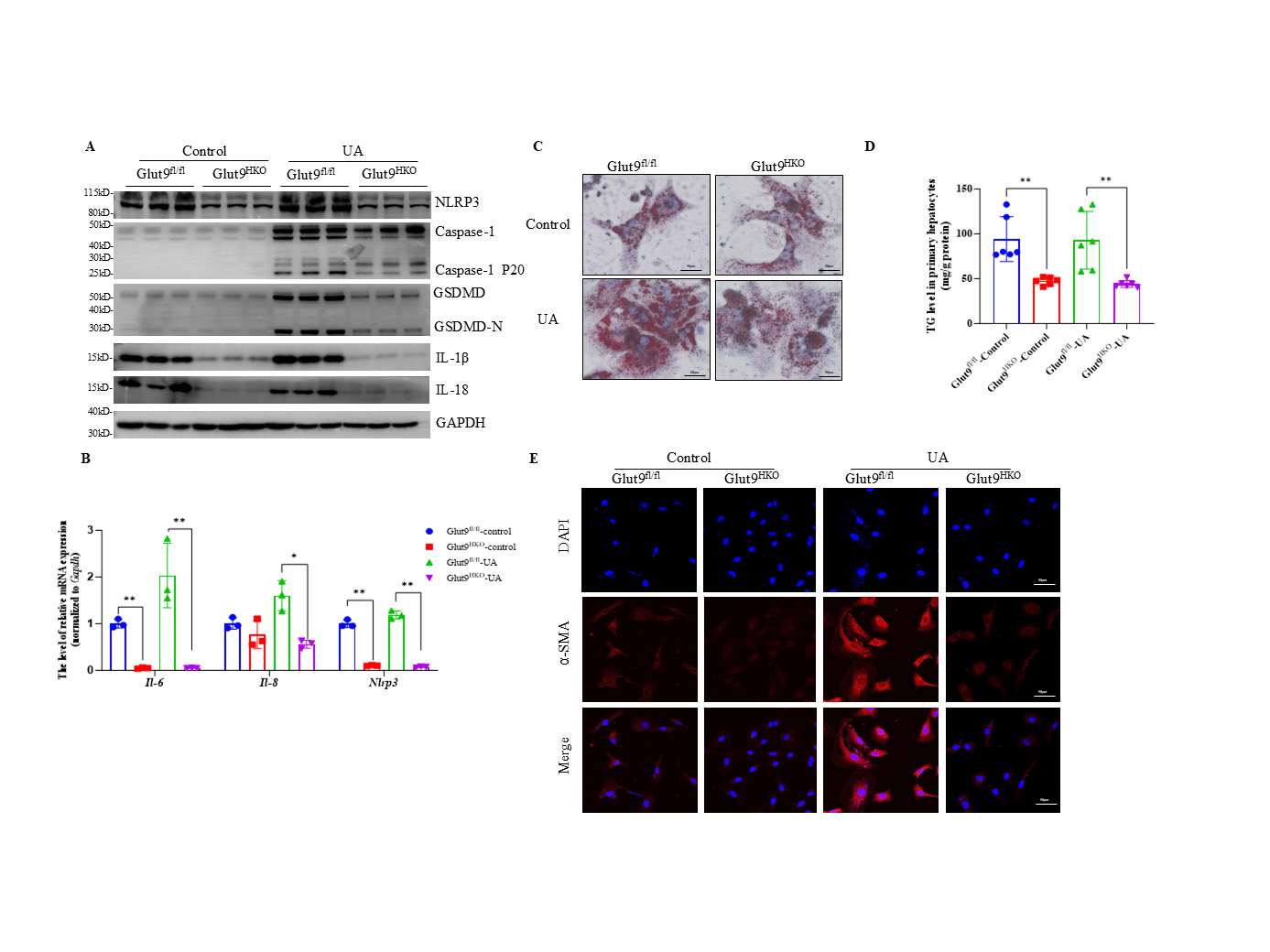
**

**Supplementary Figure 4 Glut9 hepatic knockout in hepatocytes reduced the NLRP3-Caspase-1-GSDMD pathway and decreased the activation of HSCs**

A. The protein expression of NLRP3-Caspase-1-GSDMD pathway in hepatocytes isolated from Glut9^HKO^ and Glut9^fl/fl^ with UA treatment or not.

B. The mRNA expression of *Il-6*, *Il-8*, and *Nlrp3* in hepatocytes isolated from Glut9^HKO^ and Glut9^fl/fl^ with UA treatment or not.

C-D. The ORO stain and TG content of hepatocytes isolated from Glut9^HKO^ and Glut9^fl/fl^.

E. The activation of HSCs co-cultured with primary hepatocytes isolated from Glut9^HKO^ and Glut9^fl/fl^.

Data are representative of three independent experiments with similar results. **P*<0.05, ***P*< 0.01.

**Supplementary Figure 5**

**
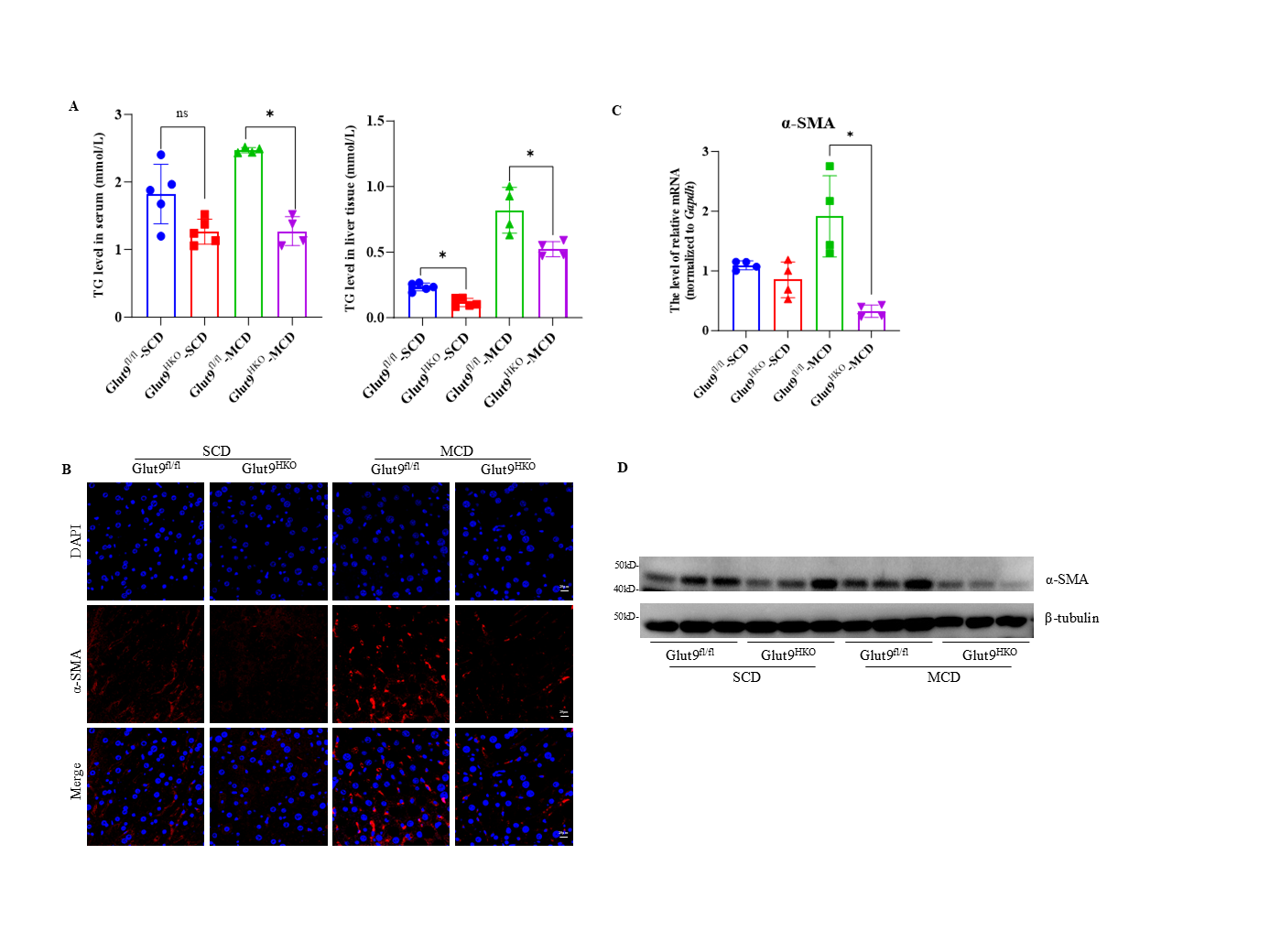
**

**Supplementary Figure 5 Glut9 liver specific knockout significantly alleviated MCD induced-NASH**

A. The TG content in serum and liver

B. The activation of HSCs was decreased in Glut9^HKO^ mice group than that of Glut9^fl/fl^ group mice whether SCD or MCD feeding.

C-D. The QPCR and Western blot showed a decreased expression of α-SMA in Glut9^HKO^ mice group.

Data are representative of three independent experiments with similar results. **P* < 0.05

**Supplementary Figure 6**

**
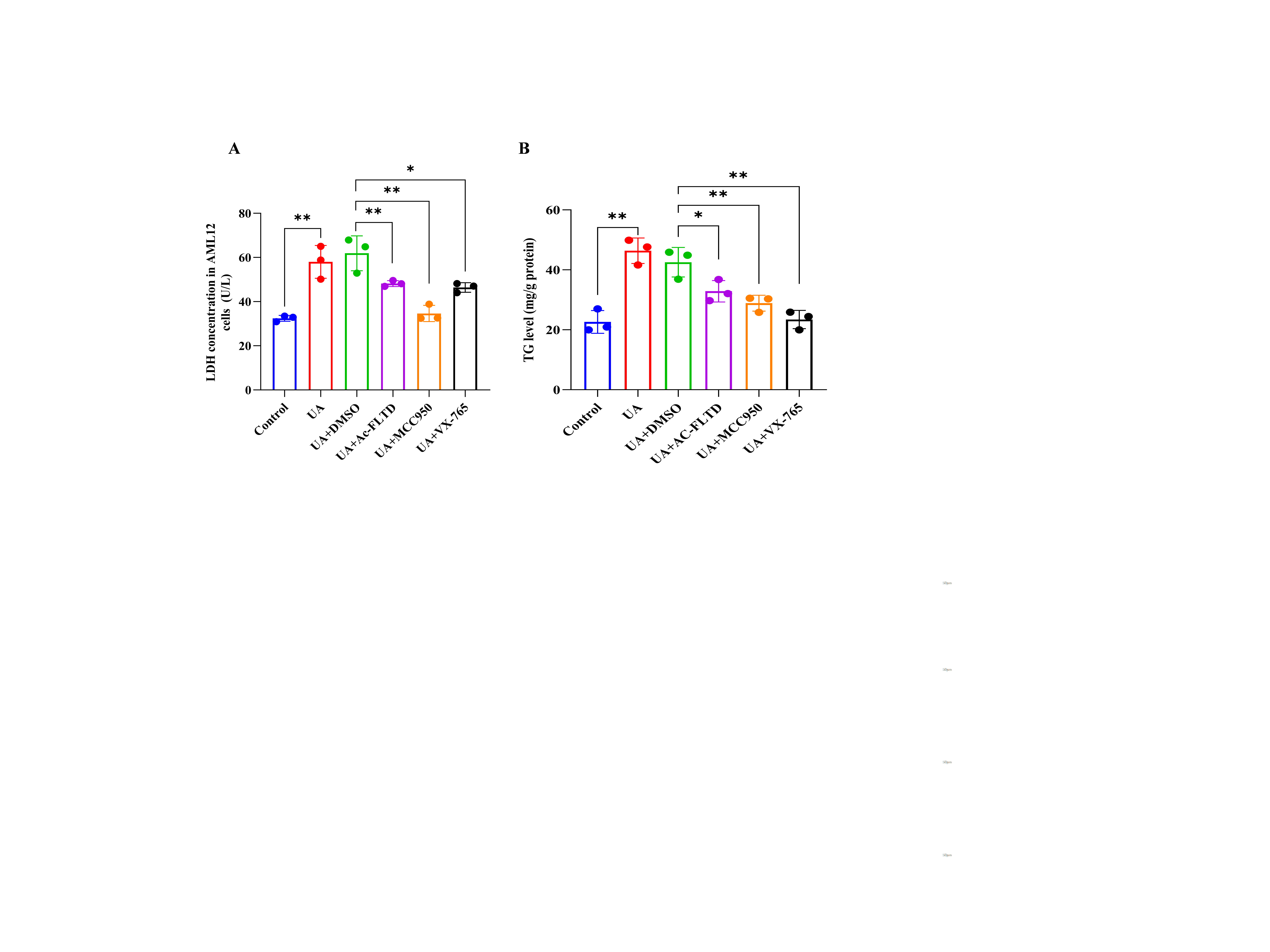
**

**Supplementary Figure 6 Inhibition of pyroptosis attenuates hepatocytes injury in vitro and reduces the weight in vivo**

A-B. The level of LDH and TG content in AML12 cells exposing to UA or different treatment.

Data are representative of three independent experiments with similar results. **P*<0.05, ***P*< 0.01.

**Supplementary Figure 7**


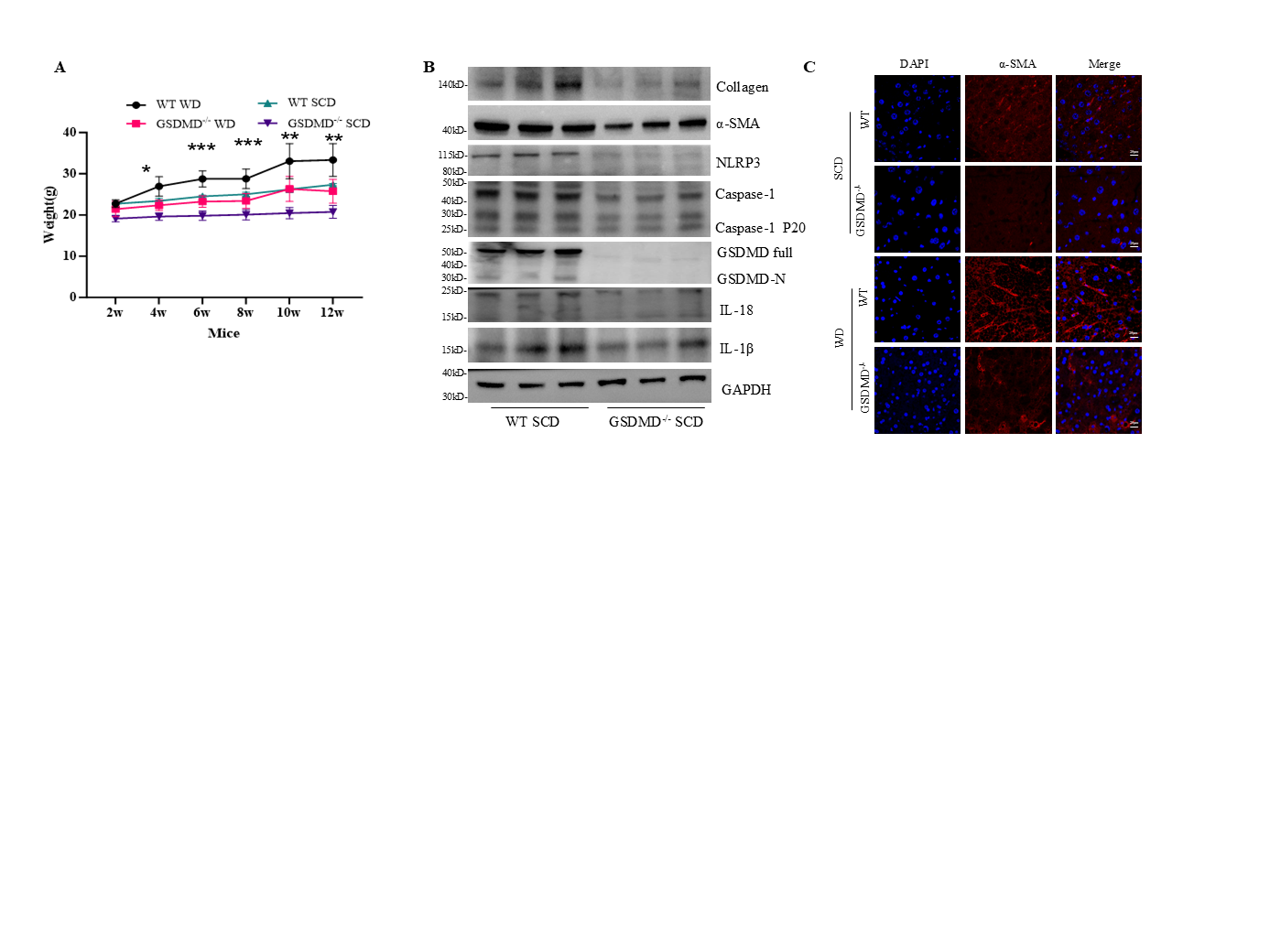


**Supplementary Figure 7 GSDMD deficiency alleviates liver inflammation and fibrosis caused by high level UA in NASH**

1. The curve of weight of GSDMD^-/-^ and WT group.
2. The expression of pyroptosis- and fibrosis- related proteins between GSDMD^-/-^ and WT group.
3. The representative image of HSCs activation of GSDMD^-/-^ and WT group.

Data are representative of three independent experiments with similar results. **P*<0.05, ***P*< 0.01, ****P*< 0.001.
